# What works in practice: user and provider perspectives on the acceptability, affordability, implementation, and impact of a family-based intervention for child overweight and obesity delivered at scale

**DOI:** 10.1186/1471-2458-14-614

**Published:** 2014-06-17

**Authors:** Patricia J Lucas, Katherine Curtis-Tyler, Lisa Arai, Sally Stapley, Jamie Fagg, Helen Roberts

**Affiliations:** 1 School for Policy Studies, University of Bristol, Bristol, UK; 2 School of Health Sciences, City University London, London, UK; 3 School of Health and Social Care, Teesside University, Middlesbrough, UK; 4 UCL Institute of Child Health, London, UK

**Keywords:** Childhood obesity, Qualitative research, Intervention, Health commissioning, Parents, Families, Local government, Health policy

## Abstract

**Background:**

As part of a study considering the impact of a child weight management programme when rolled out at scale following an RCT, this qualitative study focused on acceptability and implementation for providers and for families taking part.

**Methods:**

Participants were selected on the basis of a maximum variation sample providing a range of experiences and social contexts. Qualitative interviews were conducted with 29 professionals who commissioned or delivered the programme, and 64 individuals from 23 families in 3 English regions. Topic guides were used as a tool rather than a rule, enabling participants to construct a narrative about their experiences. Transcripts were analysed using framework analysis.

**Results:**

Practical problems such as transport, work schedules and competing demands on family time were common barriers to participation. Delivery partners often put considerable efforts into recruiting, retaining and motivating families, which increased uptake but also increased cost. Parents and providers valued skilled delivery staff. Some providers made adaptations to meet local social and cultural needs. Both providers and parents expressed concerns about long term outcomes, and how this was compromised by an obesogenic environment. Concerns about funding together with barriers to uptake and engagement could translate into barriers to commissioning. Where these barriers were not experienced, commissioners were enthusiastic about continuing the programme.

**Conclusions:**

Most families felt that they had gained something from the programme, but few felt that it had ‘worked’ for them. The demands on families including time and emotional work were experienced as difficult. For commissioners, an RCT with positive results was an important driver, but family barriers, alongside concerns about recruitment and retention, a desire for local adaptability with qualified motivated staff, and funding changes discouraged some from planning to use the intervention in future.

## Background

In common with many developed countries, the number of overweight and obese children in England is high. Accompanying this, a range of interventions are now offered to help them maintain or reduce weight [1]. Recent NICE (National Institute for Health and Care Excellence) guidance suggests that those who plan or commission child weight management services provide family-based services [1]. These include strategies to support all close family members to change their eating behaviours and increase physical activity. The guidelines suggest that services be developed with input from professionals, children and young people and their families [1]. Data from randomised controlled trials (RCTs) can provide evidence of effectiveness or otherwise in demonstration programmes, but few trials describe acceptability and take up [2]. End users of both clinical and public health research want evidence about what will work for *them* and, in the case of public health interventions, for their communities [3]. ‘Real world’ evaluations have the potential to inform commissioning decisions [4] and such decisions need to draw on the experiences and perceptions of service users [5,6].

The MEND 7–13 programme is a family-based, 10 week behaviour change intervention for children aged 7–13 who are overweight or obese^a^. Each child is accompanied by an adult/carer for two 2-hour sessions per week comprising an hour’s interactive workshop for children and parents, an hour’s exercise for the children, and an hour’s parent-only education session. Following positive results for obese children in an RCT [7], MEND was widely adopted in England and Wales as a treatment programme for overweight in children. Staff delivering the programme are generally not obesity specialists, but centralised training and resources are provided.

The work reported here forms the qualitative component of a larger study [8] which considered participation and impact when a programme is delivered at scale. The qualitative work focused on factors which might affect the uptake and implementation of MEND for both providers (particularly those responsible for deciding whether to commission MEND) and users (children and their families). We consider acceptability, affordability, perceived impact, and implementation decisions made by providers.

At the inception of this study, approximately 15,000 families had attended MEND sessions. During our study period (2011–12) MEND was provided free to families, but was not cost free. Funding came largely from Primary Care Trusts (PCTs), Local Authorities (LAs), Sport England, commercial companies, and a grant from the Big Lottery Fund (BLF). The context for both families and health services was one of uncertainty. For families the context was one of rising unemployment and, for some, cutbacks in local services. Health service re-organisation loomed large for those within the system, and the loss of the BLF funding was significant for commissioners making decisions about whether to purchase MEND (or other services) locally.

## Methods

**Figure 1:**
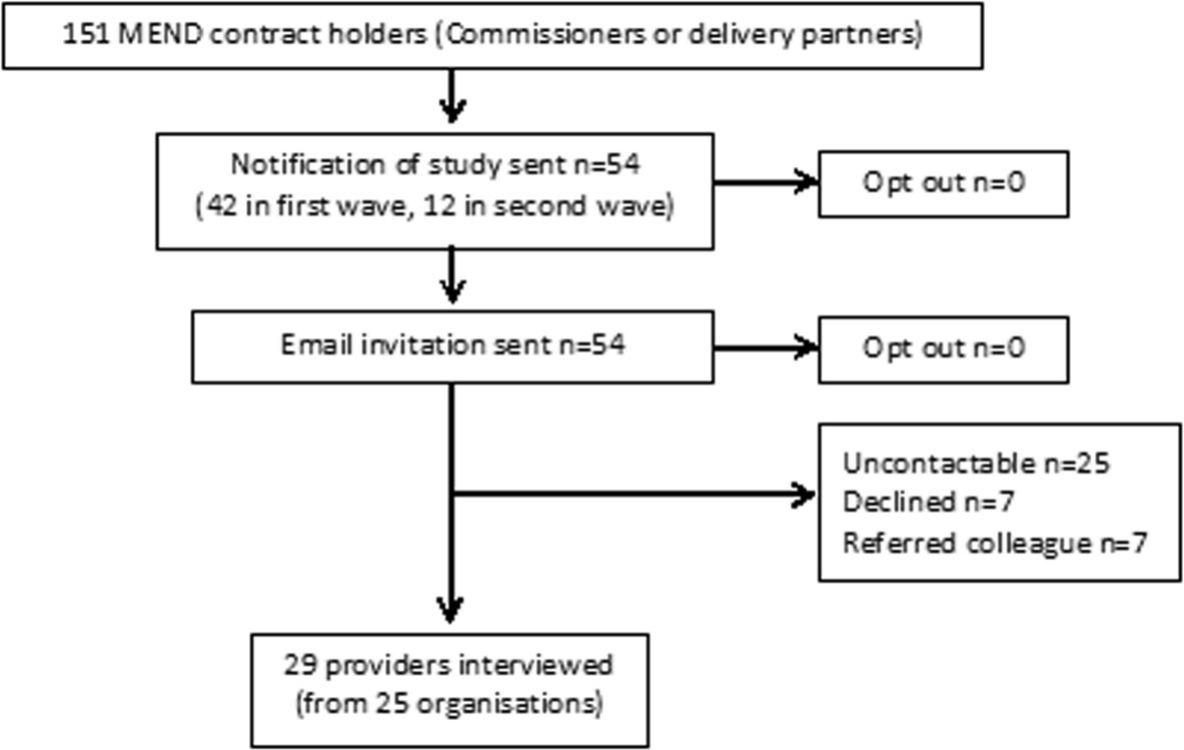
Flow chart of recruitment for provider interviews.

**Figure 2:**
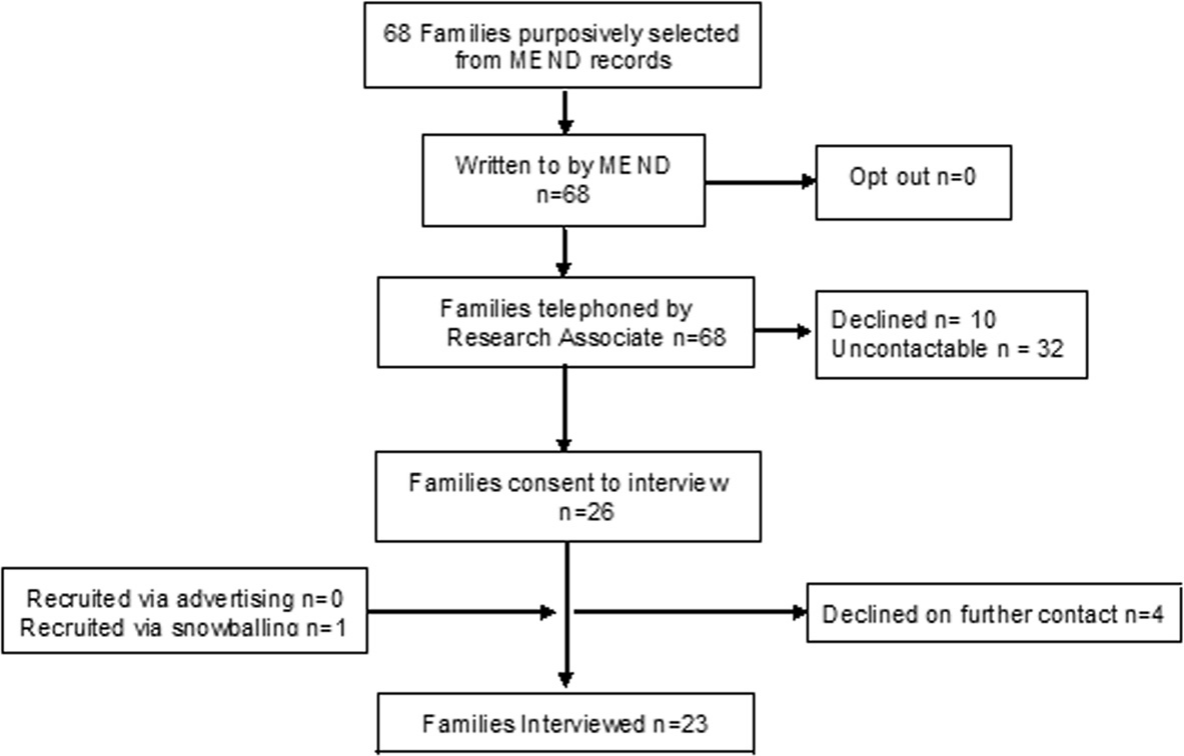
Flow chart of recruitment of families.

UCL Ethics Committee granted approval for the qualitative study with families in February 2011 (REF: 2842/001). Since many of the providers were based in the NHS, we sought NHS Research Ethics (NRES) permission. The NRES Committee deemed this component of the study to be a service evaluation not requiring NRES permission (East London Research Ethics committee REF 11/H0703/3). As a service evaluation it was also exempt from the need to seek ethics permission from UCL Ethics Committee. We conducted this component of the project with reference to the framework for research ethics produced by the Economic and Social Research Council (ESRC). Some providers (but no families) expressed concerns related to confidentiality, particularly where they had criticisms. The interview material reported here therefore has all identifiers removed; providers and families are identified by a number and letter only (P indicating Provider and F Family). Quotes are selected which illustrate or describe the themes in the words of participants, and where they afford insight into the feelings and experiences of participants. In preparation of this manuscript we have adhered to RATS guidelines [9].Our qualitative work with providers and users comprised individual and group interviews in three English regions (London, the North East and the South West). The initial approach to both commissioners and families was made by a member of the research team not, herself, involved in the interviews. We interviewed those responsible for purchasing services (commissioners) and those delivering MEND (delivery partners) together referred to as “providers” here. Our recruitment target was up to 30 providers in the expectation that we would be unlikely to generate a significant increase to our understanding after that point. A shortlist was drawn from MEND contract holders (n = 151) to achieve a maximum variation sample based on the socio-demographics of the local population (index of multiple deprivation and area ethnic composition), the type of organisation (PCT or not), and contracting details (number of programmes contracted and current contracting status. We first wrote to, and then telephoned 54 providers using contact details in the MEND database (42 in a first wave and 12 in a second wave). There were few refusals (see Figure 1), but many telephone or other contact details no longer led in the direction of the relevant person. During this period of organisational change in the NHS, we would find that people had moved on or changed post. Interviews were conducted at places or work, over the phone, or elsewhere at the request of interviewees.Our ‘user’ sample was based on family units since a parent or carer is required to accompany each child in MEND 7–13. We aimed to recruit 30 families; 10 high attenders (>75% attendance), 10 less frequent attenders (≤25%) and 10 who expressed an interest but did not take part. Anonymised MEND records (n = 657) were used to purposively select a sample of families drawn to achieve maximum variation by ethnicity, housing tenure, family structure, MEND attendance and local deprivation. Anticipating a 50% response rate, MEND Central wrote on our behalf to 68 families. Families were sent information about the study and given a 21 day opt out period after which they were telephoned up to 3 times to secure recruitment. Follow-up letters were sent to those with no working telephone number or voicemail. We subsequently introduced three supplementary methods to recruit low and non-attenders; ‘snowballing’ from the families interviewed; advertising in centres where MEND had been offered; and advertising on parent web forums. Figure 2 shows the recruitment flow for families.

Families were invited to take part in group interviews comprising the index child, the parent or carer who had attended MEND sessions and, if they wished, up to two other family members or friends they considered important to the child’s weight management, for example siblings or relatives who provided childcare. We chose to use group interviews reflecting the family approach and experience of the MEND programme. In some cases, family interviews were followed up with individual interviews designed to generate accounts which might have been unvoiced in a group interview, possibly offering an alternative narrative or prioritising voices sometimes muted in a group (as children’s sometimes are [10,11]). Most families chose to be interviewed at home, one chose a public space.

All participants were given age appropriate written and verbal information about the study prior to commencing interviews. Parents and children were asked for, and provided, written consent with the exception of one young child, who gave verbal consent.

Our topic guides were informed by the literature, our project management group and advisory groups, providers and services users including a young people’s group convened by the National Children’s Bureau. Interviews with providers covered their professional background, and role in commissioning or delivering MEND and their experiences of this, their perceptions of levers and barriers to participating in MEND (for families and providers), their local health context, and their views about the value and costs of MEND in their area. Interviews with families included; their experience of MEND, referral routes and experience of being in the programme, their views of barriers and levers to participation for families and children, their perceptions of changes to diet or health during or since MEND participation, their beliefs and feelings about weight control, and their perceptions of the costs and benefits to families and children of taking part in MEND. Initial interviews were used to pilot and refine the topic guides, which were tools rather than rules; designed to map rather than constrain discussions. Interviews were audio recorded, and sent for transcription using encrypted files and a secure file transfer system. Verbatim transcriptions were returned to the qualitative research team after removal of identifying features.

Interviews were analysed using framework analysis [12], which involves familiarisation with the data; summarising data in tables by case and low-level theme (largely drawn from interview questions); exploring emerging patterns and disconfirming data within and across cases in tabulated summaries and original transcripts; from this identifying and indexing higher order themes.

Known barriers and/or levers to effective implementation of services to promote healthy weight among obese and overweight children were considered in interpreting the data. We considered practical, programme-related, socio-cultural context and social factors [13]. Practical factors included the type and extent of funding, the accessibility and affordability of transport, staff quality and motivation, and the involvement of other agencies. Programme factors included mode of referral, implementation in local contexts, and support for participants to maintain change. These operated in combination with the degree and type of carer involvement, the interplay between practical and social aspects of the programme, and features of the community where it was implemented, including the food and built environments.

## Results

We contacted 66 providers and recruited 29 interviewees to 26 interviews (twenty four were individual interviews, with one group of 2, and one group of 3, see Figure 1). These included health and wellbeing development officers, strategic leads for obesity, medically qualified public health consultants and local programme managers or co-ordinators.We contacted 68 families through initial sampling, interviewing 22 (32%) plus 1 additional family recruited through snowballing (see Figure 2). The 23 families interviewed comprised 64 individuals; 22 mothers (including 1 foster mother), 6 fathers, 2 grandmothers, 2 aunts, 12 male and 10 female MEND attendees, 5 brothers and 4 sisters of attendees, and one family friend. By the conclusion of the majority of family interviews it was clear that interviewees had exhausted all they had to say, and only eight individual interviews took place of which two were with individuals unable to attend the family sessions.

Characteristics of families interviewed are provided in Table 1. Variation was achieved across all factors except completion status. Our sample did not include any families who had contacted but did not join a MEND programme. In the course of the study it became clear that those we recruited and interviewed on the basis of low or no attendance were mis-classified, as is frequently the case with administrative data. Families consistently reported higher attendance than MEND recorded and the one family recorded as referred but not confirmed on a MEND programme had clearly attended and had the tee-shirt to prove it. In quantitative work conducted after these samples were drawn (to be reported elsewhere) we developed a method to mitigate these issues as far as possible. The additional methods we put in place to recruit non-attenders described above were also unproductive. We know that website advertisements were read; in one location for instance, the advert was viewed 53,737 times and clicked on 26 times (though not necessarily by MEND families), but no additional (non) participants came forward.

**Table 1:** Characteristics of families Interviewed (n = 23)

		**Number of families**
Region	South West	9
	London	7
	North East	7
Year of referral	2008	10
	2009	5
	2010	7
	2011	1
Housing tenure	Owner occupier	16
	Social housing	3
	Privately rented	4
Family structure	Lone parent	11^1^
	Couple	12
Ethnicity	Bangladeshi/Bangladeshi British	2
	Pakistani/Pakistani British	4
	Other Asian	2
	Black African	1
	Black British-Carribbean	1
	White British	13
MEND attendance	Unknown	10
	25-75% recorded attendance	6
	>75% recorded attendance	7

^1^Including one foster parent.

### Costs and affordability of MEND for providers and families

Providers’ decisions to buy MEND were based on whether the programme was a good ‘fit’ with local commissioning priorities and affordable in terms of cost and budget availability. Looking forward, providers were uncertain what local authorities might value or prioritise once they became responsible for public health. Those wanting to re-commission MEND (particularly those who had received BLF funding) had concerns about budget and affordability:

“*.…at the end of the day, we can’t run [it] without a budget”.* (P39)

“*it really comes down to three…key questions for…all commissioners and providers of obesity services .. .what outcome, over what period of time, for what unit cost..*..”. (P64)

Some considered that the cost per family that *completed* the programme was high:

“*it’s an expensive programme…it has proved to be very expensive per family*”. (P10)

Providers held varying views on what they considered satisfactory programme completion/retention. For some, retaining half the families in the programme was considered sufficient, while other thought this was “*appalling*” (P10). Others felt regular attendance (for example every other week) was more important than total attendance.

Depending on their definition of ‘completion’ the perceived cost per family varied considerably. Further, providers told us that estimates of programme cost assumed that MEND training alone was sufficient, but several felt that further investment was required:

“*So to deliver it, it’s relatively straightforward because there’s a book to work from… if someone goes on the training, you don’t have to be a qualified nutritionist… but we always use a qualified nutritionist….*” (P7)

They reasoned that investing in high quality, skilled staff would be more likely to bring worthwhile returns and a more stable workforce – an aspiration supported by the national obesity support team [14]. Taking part in MEND also has costs to families. While some reported that food costs had gone down because they were eating less meat, there was less waste, and/or eating healthily could be cheap, others suggested that the healthy food recommended by MEND was a significant cost burden:

“*there isn’t a lot of money to spend on healthiest food”.* Mother (F61)

Mostly, families considered travel costs a routine part of family life rather than an addition, although a few found travel to MEND difficult where public transport was not available and driving not an option.

The greatest perceived costs for families were not financial, but the time and emotional costs. They spoke of the challenge of fitting MEND into their lives. Several parents worked shifts, often into the evening. Where sessions were organised for early evening, families were rushing to get there after school and work, and children could be tired and hungry. The needs of all the children in the family had to be considered, not just the child attending MEND. Children also had to fit MEND around other activities such as sports, after school clubs, school work and the mosque. Competition with other commitments was recognised by providers: “*it is a heck of a commitment; twice a week for two hours for 10 weeks*” (P31). As one mother put it:

“*‘My neighbour says you’re in and out of that house like a fiddler’s elbow*”. Mother (F67).

While timing issues could make participation demanding, families we spoke to readily acknowledged that finding a time that suited everyone was impossible.

Other concerns were that parents from low-income families might not be able to reconcile paid and unpaid work (including child care) with MEND:

*“.. it is also being sensitive to how much else they’ve got going on, and if you’re working with deprived families who’ve got parents working two jobs each and three or four children to manage, it’s difficult*”. (P4)

There were sometimes tensions or disagreements around the division of labour in relation to supporting children in their weight management, voiced by one father in a family discussion:

“*You had me running round the …. field. What do you mean I didn’t get involved*?” Father (F27)

For children, choosing the healthy option could mean being the odd one out. One girl (F5) described walking to school while her brother got the bus. A boy described making vegetable soup to take to school but:

*“I never ate it because of the smell. I used to … open it on the bus and they used to go, ‘Oh, what is that smell?”* Boy (F26)

Parents and children expressed their disappointment with themselves and others when they had been unable to maintain changes despite these sacrifices. One mother of three children, one with special needs, whose husband often worked away from home said:

“*I just felt like I’d invested a lot of time, forget about the money, but a lot of time and effort*.” Mother (F67)

### Provider implementation decisions

Support for MEND among providers came from the evidence-base and its readiness and availability:

“*with all the resources … they provide … MEND was kind of …already planned, already set up ready, it was easier for us .. than looking to set up a child weight management project ourselves because we just didn’t have time* ..”. (P65)

“*The main reason…was because of the RCT, I think it came out that year…that’s why we’re delivering MEND*”. (P35)

Some indicated the importance of delivering on local public sector agreements and that, as an evidence-informed programme, MEND often had at least the initial support of local PCTs. However, in considering re-commissioning, it was the perceived effectiveness of the programmes run *in their own area* was more critical than the trial results. Some commissioners were very positive:

“*everything was meticulously recorded and the results are really excellent… we’re very pleased with the outcome”.* (P48)

But others found changes in BMI small, take up low, and attrition high. There was a need expressed to gain local evidence of outcomes in the longer term:

*“why would we commission a programme that showed three or six month outcomes or even, why would we commission a programme that showed twelve month outcomes? I want a spec that shows ideally 36 month outcomes or 24 month outcomes.”* (P64)

Providers saw a tension between programme fidelity and local context. They wanted to use the best available evidence to guide decisions, drawing on several models of service for the local population. Making changes to MEND to suit the local context had been implemented by some, though not without misgivings:

“…*my initial feeling … coming from a research background, was that that’s a really bad idea because we’re not working from an evidence base. But having now been…on the coalface.., I feel slightly more inclined to understand…why people do that… [T]hey…know their own population… their delivery team. They want to give people the chance to use those skills and make sure what they’re delivering is useful in their own locality*”. (P5)

Adaptations were referred to in terms of meeting the needs of the local population, and tended to be small-scale. Modifications included suspending programmes during school holidays; ‘breaking down’ MEND materials for groups with poor literacy and adapting sessions depending on the group interaction. Translation and visual adaptation of materials were critical in one area:

“..*we have a culturally diverse community…so what we do is tailor our programmes … we use different languages. It just depends on who you’re seeing really…we try and do things to make them visual. So that is how we try and tailor things*”. (P48)

Similarly, in one locality where most families used local markets for shopping, participants were encouraged to discuss their preferred diets. In one area, more “Asian” foods were added to the list of foods used. Some families commented on the suitability of MEND materials, with one suggesting that the materials weren’t appropriate for those who could not read or write and that they all got “fed up” with the paperwork (F28).

Responding to the challenges for families in completing the MEND programme, one reported encouraging families to return to programmes to work on what they had missed:

“*But that’s not recommended practice really is it for what we do? But we’re working with real people with real lives here…sometimes what’s dreamt up in academia doesn’t…fit with working, with real people in the community*”. (P31)

Some reported commissioning alternatives or services running in parallel to address perceived shortcomings

“*MEND is not rocket science…there are about three or four different products on the market*”. (P15)

Frustrated at low recruitment and retention of families (and staff), one area implemented a ‘rolling programme’ that new families could join at any point.

*“I wanted to look at how we could make a better model to better use our staff. I feel we’ve done that. We still have the problems, but the issue I had with MEND was there was a start, there was a middle, and there was an end. So you started a programme, and then you started a new programme with new people. You couldn’t feed new people in all the way through, whereas what we’ve got is a rolling programme so we can have new families joining us all the time. While I see there’re strengths and weaknesses to both, I feel that’s a better option because I’m not then running programmes with just one or two families in them.”* (P47)

### Training and motivation of staff

The personal qualities of the MEND delivery staff (known as leaders) were important to families. Often parents would stress that leaders were nice people - “*marvellous*” Grandmother (F57); “*fabulous*” Mother (F21) and children often told us they “*made it fun*” Boy (F51). But this did not necessarily translate to a perception of professional competence. Several suggested that they did not have the necessary skills or knowledge:

“*They were dead enthusiastic. I personally didn’t think they knew as much maybe as…they should have*”. Older sister (F28)

“*I’m not so sure the person had actually gone through the package in any great depth…It felt like she was just reading … She was a lovely lady*”. Mother (F27)

Some felt that leaders lacked skills in managing a group of children. There was also a concern if leaders did not have the life experience to help. Having a facilitator who was a parent was seen as an advantage, and descriptions of some leaders as ‘*thin*’ or in one case “*looking anorexic*” Mother (F67) suggest that some attendees felt that personal experience of healthy weight management was an advantage.

Providers emphasized the importance of a skilled delivery team, an issue sometimes affected by staff turnover:

“*a lot of instructors did not…stay around for that long so we were continuingly training new instructors*”. (P52)

One commissioner insisted that MEND delivery staff received training in child protection, and others spoke about the complexities of the issues that families referred to MEND had sometimes experienced:

“*when you’re dealing with families who have multiple difficulties in their life… those difficulties… appear in the room. And that is quite a strain on the people who are trying to run those programmes*”. (P40)

### Family engagement

Providers valued the active involvement of parents and carers and saw a family approach as crucial:

“*I think that’s key… because if you don’t change the parents, then nothing changes at home…”* (P14)

They recognised that this brought challenges for recruitment. We describe challenges to retention above, which was often reported as poor, and frequently attributed to difficulties recruiting from the most deprived groups or neighbourhoods. It was suggested that the multiple problems of deprivation meant that they were less likely to engage with a programme such as MEND:

“*it is very hard sometimes to engage parents…. You’ve got [name] estate, which is one of the top 4% of council estates in the country for deprivation. There are low income families, single parent families, and there are a lot of kids in care*”. (P39)

*“obviously they came with a bundle of additional problems… language, mental health issues, they’d been abused, they’d come to a new country and tried to settle in…It’s less around budgeting and balancing your food, it’s more about feeding your kids and getting your kids clothed and staying in the country*”. (P28)

Some providers felt that there was a stigma to attending a weight management programme which might be viewed as a “Fat Club”:

“*with any programme there are always going to be stigmas*”. (P38).

Families did not explicitly describe stigma (although our failure to recruit non-attenders means this may not be typical), but several children told us they were reluctant to attend because they felt they weren’t ‘fat’ or because they resented being identified as ‘fat’:

*“You said, ‘What’s it going to be like in there?’ And I said, ‘I have no idea. I don’t know what it’s going to be like in there.’ …And then you said, ‘Is it going to be full of fat kids, mum?’”.* (Mother F70 talking to son)

For families that attended, the shared experience reduced feelings of isolation and parents were grateful to have something to turn to. Many parents valued the social acceptance of a group describing shared problems, knowing that you’re not the only one (and some are worse off than you). Some children enjoyed being able to exercise with those of similar weight.

*“I found them fun because I was surrounded by different people who were in the situation that I was in, in terms of being overweight and finding exercise difficult.”* (Girl F25)

*“to a certain extent I quite enjoyed the sessions. There’s different people that you’re interacting with”.* (Father F4)

As one provider said of parents:

“*they like the fact that they’re in a forum where they can speak to other parents who are like-minded or are going through similar circumstances*”. (P54).

For families we interviewed who attended MEND, engagement with the process was high. Most had self-referred, having seen leaflets or heard about MEND from friends or family. This was usually at the instigation of the mother who was worried about overweight (although parents seldom explicitly used the term). The most common reason given for attending was that children had experienced bullying or social isolation and parents and children felt that losing weight might reduce this:

*“I used to get bullied a lot”.* (Boy F18)

This resonates with children’s focus on the social impact of overweight reported in other studies [15-17]. In our study, this was often associated with the transition to secondary school. This was viewed as an important moment because of the fear or experience of bullying at the “*teenage school*” (Mother F61). There was a sense that this was a time either to capitalise on changes for the better, or to become entrenched in bad habits.

Mothers seemed more engaged than fathers. The only family where a mother was not behind enrolment in MEND was a father parenting alone. Where children lived with both parents, the mother accompanied the child to MEND in all but one family. Fathers attended occasional sessions, and other family members helped out; grandmothers, aunts and older sisters attended when parents could not. It was common for younger siblings to attend alongside the child attending MEND if no other childcare was available.

### The context and wider environment

Providers were well aware of the links between obesity, deprivation and ethnicity. The socio-demographic profiles of the areas where MEND had been implemented varied with urban and rural areas, affluent and poor neighbourhoods. Local recruitment efforts targeted districts where child obesity levels were known to be high, and obesogenic aspects of the locality were discussed, including the high number of fast food outlets, lack of access to outdoor play, lack of public funding for facilities such as swimming pools, and poor public transport, particularly in rural areas. Other factors raised by providers were a lack of jobs, and high levels of depression, alcohol abuse and domestic violence. Understanding the profile of the local populations where MEND was being implemented was seen as key to recruitment and addressing inequalities in childhood overweight.

Many families felt that the choices that children could make were constrained by their environments; that neighbourhoods weren’t necessarily safe, and exercise activities inaccessible:

“*I don’t really feel too safe going out on my own just doing stuff especially on a bike because I have a BMX but I don’t want it to get robbed*…” Boy (F14)

One child who could only walk short distances with support lived in a built-up area with major roads and few crossings, For another family, moving to a new area meant using a school bus and father and daughter spoke of their disappointment that she could not join after school clubs that might have helped her to be more physically active.

Parents and children described the temptations of fast food in their environments, and the irony of other kinds of commercial sponsorship, underlining the point made by Hastings on the harmful consequences for public health of marketing [18]:

*“… And they’re on about the obesity epidemic but they’ll take on, say, McDonalds instead of Nike or Adidas to actually support those [sporting] facilities.”* Aunt (F57)

Families from all backgrounds spoke about rich food being associated with festivals and celebrations, and these being difficult times to keep to a healthy diet. Grandparents wanted to ‘treat’ their grandchildren by giving them sweets and rich foods. Some participants whose families had their origins in low income countries drew a contrast between an obesogenic environment in England and a context where, despite fewer resources, a healthy life could be achieved:

‘*my great granddad…the day before he died he was still working on the fields*’. (Father F51)

We asked providers whether they felt commissioning or participating in MEND created any knock-on effects in attempting to mitigate an obesogenic context. Only one offered an example:

“*I’m not aware of anything actually chang[ing].. But … parents have been quite, sort of shocked, when they speak to the nutritionist and … get from their children, what they really eat in school… several of them have said, well, I’m going to take this up with the head teacher…. And some of them have actually questioned how little physical exercise there is on at school, but whether or not anything has actually changed from that, I don’t know*.” (P7)

### Perceived impact and maintaining change

Most families reported having gained something positive from the experience of participation in MEND, enjoying particular sessions, having fun, meeting others. However few felt MEND had made a significant contribution to weight management and tended to attribute any long- term change in weight to other factors.

Participants spoke of MEND as taking place at a particular point in a family’s life and easy to leave behind. They spoke of a desire to move on, to return to ‘normal’. Many reported keeping to MEND changes as much as possible, but reverting to unhealthy options (particularly takeaways) at busy, or “special” times such as when they are on holiday from school or “*if people are coming over*” Mother (F56).

“*you’re running around … trying to run a house, keep a job down, send the kids everywhere, pick them up, doing all that, then you think ‘I’m starving and you just grab something*.” Mother (F26)

Changing family circumstances contributed to this. Parents commit to MEND when they sign up, but families are not static. It can be a challenge not to fall “*back onto the usual*” (Daughter F5). Over the period since they had first contacted MEND, families in our sample had experienced divorces, deaths, births, moving jobs and homes. It could be difficult to retain the changes MEND recommended. Many had completed MEND some time before our interviews in 2011 and 2012 (see starting dates in Table 1) which may have contributed to a MEND being a distant memory.

Context could change in positive ways too. Where long term change in weight status had been achieved, this had tended to coincide with an important life transition for the child, including moving to senior school, a teachers’ interest, new opportunities for play, or simply getting older and having more independence:

“*I think it was just a combination of everything. We changed how he ate. And then he started walking to school and back, and now he’s got the dog*.” Mother (F18)

Children liked being recognised as decision-makers about their eating as others have described [19], and being given control of shopping and cooking was described as key to successful weight management for some. Maintaining change required willingness not just from the child but the whole family to sustain the personal cost of giving up favoured foods or activities and taking on new, possibly less favoured foods or activities:

“*I think most things happened after the MEND programme. They put in your head what you need to do, and it’s up to you to follow it through. And being on the MEND programme twice a week was enough to concentrate on while we were on the programme. So the rest… we kind of got the knowledge, and then took it away and did something with it*.” Mother (F18)

Family dynamics are important because parents may use food to reward good behaviour [20]. Conflict in families can result in ambivalence towards weight management [21] and in our study, conflicts across the generations were often clear: 

“*She [mother] still has white bread, don’t you…Which means then [daughter] technically gets white bread because that’s what mam likes*” Sister (F28).

Although participants had found it difficult to find time for MEND, once the programme was over, they missed the support of a “safe” group. The few families who had attended follow up activities set up locally had been disappointed that none of the families they knew were there. Few had logged onto the MEND website and in any case, providing a screen-based format was seen to contradict MEND advice:

“*There we are exercising twice a week and they say, “go and sit at a computer”. I just couldn't get my head round that at the time*”. Mother (F67)

## Discussion

Dealing with overweight and obesity is complex, as the Foresight report [22] makes clear. The observations here describe family and provider views on treatment implementation. Our findings confirm the levers and barriers identified in our earlier mapping study [13]. Practical problems such as transport, parents’ work schedules and competing demands on family time were common. All the families we spoke to found these difficult, but there were particular pressures for low-income parents, and this may have implications for access. The providers of this programme clearly worked hard to deliver in low income areas, but in order to optimise the ‘reach’ of such programmes, some of the issues described in the findings here need to be addressed. Delivery partners often put considerable effort into recruiting, retaining and motivating families, which increased uptake but also increased cost. Parents and providers valued highly trained delivery staff, again impacting on costs, and providers often felt the need to make small adaptations to local social and cultural needs. Both providers and parents expressed concerns about the long-term success of the programme, and the way that this could be compromised by an obesogenic environment. In many areas barriers to use, together with concerns about funding, created barriers to commissioning. Where these barriers were not experienced, and where the scheme was perceived as effective locally, commissioners were enthusiastic about continuing to fund MEND.

Most families reported having gained something positive from the experience of participation. However, it was often difficult to sustain the changes associated with the intervention in the longer term. The constraints imposed by the wider environment, the fact that family life was changeable, or simply the ‘pull’ of established ways of life made it hard. Families’ concerns about sustainability were shared by providers who felt that MEND was supportive while it was running, but that children and families needed further support to persevere in the longer term. MEND 7–13 is a 10 week programme with short term follow up. Long term improvement is known to be hard to achieve [23]. A plausible argument has been made [24] that improvements in child weight management are more likely to take place in families who are engaged and ready to make changes. Among the families we spoke to, living in circumstances which facilitated change (for instance having the time, space and assets to get to and from the programme) was also crucial, as were ‘pilot lights’ for change such as a new school, or a dog with his own exercise requirements.

MEND families have been interviewed in three other research studies to our knowledge. These explore views of MEND delivered through primary care [24], views while attending sessions [25], and choices between interventions [26]. Like us, Turner [24] found parents wanted advice from someone who they felt had both the professional and personal experience to understand the difficulties they faced. Staniford and colleagues [25] interviewed families and professionals with experience of a range of obesity treatments. In their study, professionals were disappointed about attrition and lack of long term weight change but also frustrated that families did not become ‘independent’ at the close of the programme. The authors noted that:

“*By teaching behaviour change/weight control techniques in a contextual vacuum, participants are highly likely to remain vulnerable to the same environmental influences*” [25] p. 240.

Parenting is an onerous job, which many combine with jobs and job-seeking. Whilst activities within MEND (e.g. supermarket visits and exercise) take some account of context, without wider action on the determinants, creating and maintaining healthy weight may simply be too much. As a community participant in another context tells us, focussing largely on individual behaviour in an unsafe environment can be “like teaching children to swim in a pool full of alligators” [27] p. 730.

None of these studies contacted non-attenders, and a major limitation of our study was our inability to interview those who had been in touch with MEND but never joined a programme (‘refusers’), or who attended once or only a few sessions (≤25%). This is not a unique failing, a similar study of a family-based child obesity intervention also had low response rates from non-participants [28]. We had reason to be positive about our ability to recruit refusers, having successfully done so following an RCT in the past [3]. Time and interest are likely barriers to responding, but we believe these were exacerbated by two impediments in this study. One was the unexpected sampling challenge associated with service records. Secondly (particularly for busy families), changes to research governance structures meant that we had to take a lengthy route to contact ‘refusers’ in contrast to the more direct methods a decade earlier. Despite this gap in our sample, the ‘good’ attenders we talked to were eloquent in telling us what had made attending and engaging with MEND hard as well as what had ‘worked’ for them.

Methods for understanding and evaluating public health interventions such as MEND which take place in complex social and economic settings are still in their infancy. Influential in terms of theory have been Hawe and colleagues [29,30] on local context and interventions as events in systems. In terms of methods, the Cochrane Public Health Review Group [31] has given encouragement to methodological plurality, equity, and attention to users. Guidance on complex interventions and natural experiments have propelled the field forward [6,32] and funding for robust public health research has increased. In this context it is important to ensure that research results are not viewed as commodities providing simple solutions to complex problems. Implementation issues such engagement, local context, staffing, appropriateness of intervention content, funding constraints and commissioning policies identified here will be common to many public health interventions. The most recent NICE guidance points to the importance of addressing these, particularly the impact of short term funding streams, when developing services for managing childhood obesity [1].

In this study, a good deal of significance was attached by providers to the positive results of an RCT but, as several pointed out, context – geographical, political and cultural - matters. This adds a further layer of complexity for those wanting to implement evidence-informed programmes. In their analysis of the need for a joined up approach to public health planning for childhood obesity, Hendriks and colleagues point to harsh treatment of interventions which admit to problems, a lack of learning by doing, and a lack of interest in implementation as part of the planning and policy process [33]. Reporting problems and difficulties is counter-cultural, and the norm is to disseminate stories of success rather than learning from what goes wrong. The providers we spoke with clearly felt ‘pinched’ by organisational behaviours which required solutions validated in a research context, but where strong applicability to local context was also needed.

Finally, our interviews were with commissioners/providers and families and not with our colleagues who developed MEND and who also, of course, have expert views. They did not always feel that the perceptions of families and commissioners were correct or fair. However, these perceptions and experiences are among the factors that those implementing weight management programmes take into account.

## Conclusion

This article sets out qualitative findings in relation to the acceptability, affordability, implementation, and impact of a treatment programme for overweight and obese children. Most families felt that they had gained something, but despite most of those interviewed being good attenders and to that extent well-disposed to the programme, few expressed the view that it had ‘worked’ for them.

For commissioners, an RCT with positive results was an important driver for implementation, but family barriers, alongside concerns about cost and long term success discouraged some from planning to use the intervention in future.

There is a lack of evidence to support decisions about which service to provide for overweight children. Interventions with significant external funding may struggle once that funding comes to an end if commissioners are unsure of cost effectiveness. At a population level, the biggest and most lasting public health gains are likely to be achieved by acting on obesogenic environments [34]. As the families in this study describe, this environment creates difficulties for individuals attempting change. The move of public health into local authorities in the United Kingdom, even in a climate of austerity, may present new opportunities to act on determinants including obesogenic factors, as well as on behaviours through treatment programmes.

## Endnote

^a^Online support is available for 2 years after completion.

## Competing interests

We declare no financial relationships with any organisations that might have an interest in the submitted work. We declare a non-financial relationship with MEND Central, who were part of the project advisory group and provided access to data and participants. Given that results from the project might support, threaten or have no effect on MEND Central’s success, our grant application and subsequent protocol set out governance measures to ensure that any competing interests, and the interests of project stakeholders, were managed appropriately. We declare no other relationships or activities that could appear to have influenced the submitted work**.**

## Authors’ contributions

HR conceived and designed the qualitative study, with subsequent design contributions from PL, KCT and LA. HR, PL, KCT, LA and SS conducted the interviews and analysed the data, and all authors contributed to interpretation of data. PL and HR drafted the article and all authors revised it. All authors approve the final version of the article. HR is guarantor of the study.

## Pre-publication history

The pre-publication history for this paper can be accessed here:

http://www.biomedcentral.com/1471-2458/14/614/prepub
